# Bioaccumulation of Heavy Metals by Clarias gariepinus (African Catfish) in Asa River, Ilorin, Kwara State

**DOI:** 10.5696/2156-9614-9.21.190303

**Published:** 2019-03-14

**Authors:** Olaniyi Afolabi Opasola, Adedotun Timothy Adeolu, Ayodeji Yusuf Iyanda, Solomon Olayinka Adewoye, Sunday Asabi Olawale

**Affiliations:** 1 Department of Environmental Management and Toxicology, Kwara State University, Malete, Nigeria; 2 Department of Environmental Health Science, Kwara State University, Malete, Nigeria; 3 Department of Pure and Applied Biology, Ladoke Akintola University of Technology, Ogbomoso Oyo State, Nigeria; 4 Department of Environmental Health, College of Health Technology, Ilesa, Osun State, Nigeria

**Keywords:** River Asa, bioaccumulation, heavy metals, *Clarias gariepinus*, Ilorin

## Abstract

**Background.:**

Harmful wastes and other dangerous industrial by-products constitute major sources of environmental pollutants in Nigeria. Industrial pollutants discharged into the environment contain organic and inorganic pollutants in dissolved, suspended and insoluble forms. Fishes are known for their innate potential to bioaccumulate heavy metals in their muscles and various organs.

**Objectives.:**

The present study aimed to assess the bioaccumulation status of heavy metals in selected organs and tissues of African catfish in Asa River, Ilorin, Kwara State, Nigeria.

**Methods.:**

Three sampling points (A (upstream), B (point of discharge) and C (downstream)) were selected in relation to industrial effluents that enter the river. African catfish were randomly harvested from each site using fishing nets at the three sampling points. The samples were digested and subjected to atomic absorption spectrophotometric analysis. Statistical analysis of data was carried out using the Statistical Package for the Social Sciences (SPSS) version 20 and two-way analysis of variance (ANOVA) to compare data among sites and organs.

**Results.:**

The present study found that the bioaccumulation level of heavy metals in selected organs and tissues of African catfish in Asa River, Ilorin, Kwara State, Nigeria was very high and the level of accumulation of heavy metals increased downstream. Accumulations were much higher during the dry season (especially in the month of February) and relatively low during the peak of the rainy season.

**Conclusions.:**

Heavy metals at elevated levels in the aquatic environment can accumulate in fish tissues and organ, and therefore fish from the Asa River are not fit for human consumption.

**Competing Interests.:**

The authors declare no competing financial interests.

## Introduction

In Nigeria and throughout the globe, industries are crucial to economic development and play an important role in raising living standards. Pollution of the aquatic environment with heavy metals has become a worldwide problem because the metals are indestructible and most have toxic effects on organisms.^1–3^ Heavy metals enter rivers and lakes from a variety of sources such as rock and soil directly exposed to surface water, in addition to the discharge of treated and untreated liquid wastes into water bodies. These harmful wastes and other dangerous industrial by-products constitute major sources of environmental pollutants.^2^ Most industrial pollutants discharged into the environment contain organic and inorganic pollutants in dissolved, suspended and insoluble forms.^3^,^4^ Effluents discharged into the water bodies may affect fish and other aquatic organisms, either directly or indirectly. Most rivers and freshwater streams are seriously polluted by industrial waste water discharged from factories. A large amount of water used in industry turns into wastewater that pollutes surface and groundwater, posing health hazards. Heavy metal ions do not degrade into harmless end products and are toxic to humans and the surrounding environment.^5^,^6^

The African catfish is a large, eel-like fish, usually of gray or black coloration on the back, fading to a white belly. It is nocturnal, like many catfish. It feeds on both living and dead animal matter. Owing to its wide mouth, it is able to swallow relatively large prey whole. It is also able to crawl on dry ground to escape drying pools and survive in shallow mud for long periods of time between rainy seasons.^7^ The demand for fish and shellfish products has increased with a growing human population and increased food supply demand. People worldwide obtain about 25% of their animal protein from fish and shellfish.^8^

Fishes are known for their innate potential to bioaccumulate heavy metals in their muscles and organs. Studies have examined the accumulation of metals in the tissues of fish from aquatic environments due to increased demand for fish as a protein source.^9^,^10^ Due to their toxicity and accumulation in the biota, determination of the levels of heavy metals in fish species has received increased attention globally.^11^,^12^ However, there is a paucity of information on the bioaccumulation of metals in African catfish from different sites along the Asa River. The present study aims to assess the bioaccumulation status of heavy metals in African catfish from three sampling sites in Asa River, Ilorin, Kwara State, Nigeria.

## Methods

The city of Ilorin lies on latitude North 8° 30′ and longitude East 4° 35′ near the southern fringe of the savannah and forest zone. It had a population of 777,667 in the 2006 census. It is surrounded by a wall about 10 miles in circumference and as high as 20 feet in some places. A large part of the province is located on grass plains with undulating landscapes which are well watered and highly agricultural. At the southern Nigeria provincial borders, at an elevation of 1,500 feet, there is a watershed with a river generally running from west to east and flowing into the River Niger. The ecology of the region plays an important role in the area's settlement patterns. It has a mean annual rainfall of 1,318 mm (51.9 inches), which allow inhabitants to practice arable farming. The mild climate has also attracted northern pastoralists to the region. Ilorin city is the commercial and administrative centre of Kwara State. It is made up of four local government areas (Ilorin South, Ilorin West, Ilorin East and Asa).

The Asa River has a surface area of 302 hectares and a maximum depth of 14 m, located approximately 4 km South of Ilorin, township Kwara State, Nigeria.^13^ The river lies between latitude 8° 28N and 8° 52 N and longitude 4° 45° E. Many residents of Ilorin and its environs depend on this river as their major source of water for agricultural activities such as irrigation of farmland and fishing. Three sampling points (A, B and C) were selected in this study in relation to industrial (soap and detergent) effluents that enter the stream. Point A is upstream along Osere stream that links the Asa River, about 1.5 m from the discharge point of industrial effluent (point B). It occasionally receives refuse and runoff water from land and other effluents, while point C is downstream of Osere stream. The choice of the sampling points was based on the accessibility, the rate at which they receive effluents from soap and detergent industry, the extent of their pollution and distance from the Global Soap and Detergent Industries manufacturing plant.

### Sample collection

African catfish (mean weight 129.4±1 g and mean length 25.2±2.00 cm) were randomly harvested using fishing nets to catch fish from three points (A= upstream, B= point of discharge and C= downstream). There were no fish found at Point B throughout the sampling period. Ten (10) fishes caught from sites A and C were stored in clean coolers packed with ice blocks in order to maintain optimum temperature and immediately taken to the laboratory for dissection using stainless steel scalpels. The sampling period was January (peak of dry season) to September (a period of continuous rainfall) in 2018. The fish organs (gills, head region, bone and muscles) were selected based on their ability to bioaccumulate and biomagnify pollutants in the aquatic environment. The fish organs were dried separately for 24 hours to a constant weight in an oven at 80°C. Each dried sample was pooled and milled with mortar and pestle. They were kept in dry labeled foiled paper and stored in desiccators until digestion.

### Acid digestion of samples

The samples were digested by adding 3 ml of nitric acid (65%) and 1 ml hydrogen peroxide (35%) as described by Taghipoura and Aziz.^14^ Hydrogen peroxide was added to the nitric acid as it reduces nitrous vapors and accelerates the digestion of organic matter by raising the temperature.^15^ The microwave was adjusted for 20 mins at 150°C and samples were then left for 35 mins to cool in the microwave until they reached room temperature. The samples were then transferred to clean volumetric flasks and diluted to 50 ml with deionized water. Samples were filtered using Whatman filter paper. Concentrations of selected heavy metals including zinc (Zn), manganese (Mn), iron (Fe), lead (Pb), copper (Cu), cadmium (Cd) and chromium (Cr) were then determined using an atomic absorption spectrophotometer (Unicam 969, Analytical Technology Inc., Cambridge, United Kingdom). Statistical analysis of data was carried out using the Statistical Package for Social Science (SPSS) version 20 software program and two-way analysis of variance (ANOVA) to compare data among sites and organs at a p<0.05 level of significance.

## Results

The mean and statistical comparisons of Zn, Mn, Fe, Pb, Cu, Cd and Cr concentrations in the gill, head region, bones and muscles of Clarias gariepinus from the sampling sites in River Asa are shown in Tables 1–7. Concentrations of Zn, Mn, Fe and Pb varied significantly across the four tissues with the exception of head regions and bones across all months and sampling sites examined. High concentrations of Zn, Mn, Fe and Pb were recorded in fish samples from Site C in the month of February, while the lowest concentrations were found in the month of September. Gill tissues had the highest concentrations of metals followed by bones, head regions and muscles. The bioaccumulation of metals in the tissues of African catfish from Site A and C was in the order of gills > bones> head regions and muscles.

**Table 1:** Mean Monthly Value of Zinc in Selected Organs of Adult C. gariepinus from Points A and C (mgkg^−1^)

	January	February	March	April	May	June	July	August	September

**Point A**									

G	1.32 ± 0.52a	1.40 ± 0.13a	1.28 ± 0.25a	1.16 ± 0.41a	1.02 ± 0.60a	0.98 ± 0.15a	0.98 ± 0.63a	0.72 ± 0.22a	0.67 ± 0.61a
H	0.83 ± 0.42b	0.91 ± 0.15b	0.86 ± 0.31b	0.78 ± 0.19b	0.72 ± 0.14b	0.63 ± 0.10b	0.61 ± 0.12b	0.43 ± 0.11b	0.41 ± 0.43b
B	1.02 ± 0.28b	1.16 ± 0.20b	1.07 ± 0.25b	0.92 ± 0.10b	0.88 ± 0.78b	0.70 ± 0.19b	0.68 ± 0.10b	0.56 ± 0.20b	0.48 ± 0.19b
M	0.66 ± 0.30c	0.79 ± 0.41c	0.63 ± 0.21c	0.52 ± 0.21c	0.46 ± 0.20c	0.40 ± 0.11c	0.36 ± 0.15c	0.30 ± 0.43c	0.25 ± 0.10c

**Point C**									

G	2.55 ± 0.31a	2.81 ± 0.20a	2.28 ± 0.18a	1.81 ± 0.24a	1.55 ± 0.10a	1.48 ± 0.53a	1.50 ± 0.45a	1.36 ± 0.43a	1.36 ± 0.53a
H	1.80 ± 0.24b	1.62 ± 0.24b	1.30 ± 0.15b	1.22 ± 0.45b	1.10 ± 0.17b	1.08 ± 0.31b	1.09 ± 0.20b	0.98 ± 0.20b	1.08 ± 0.20b
B	1.72 ± 0.23b	1.50 ± 0.30b	1.19 ± 0.10b	1.20 ± 0.10b	1.09 ± 0.11b	1.09 ± 0.19b	1.10 ± 0.15b	0.95 ± 0.11b	1.03 ± 0.31b
M	0.60 ± 0.12c	0.90 ± 0.14c	0.90 ± 0.13c	0.99 ± 0.22c	0.60 ± 0.21c	0.90 ± 0.33c	0.80 ± 0.22c	0.50 ± 0.24c	0.60 ± 0.05c

Abbreviations: G, Gill; H, Head region; B, Bone; M, Muscle.

All data are mean ± standard deviation of triplicate determinations.

a, b, c indicate means from lowest to highest. Means in the same row with the same letter are not significantly different. Means within the same row with different letters are significantly different at p<0.05.

**Table 2:** Mean Monthly Value of Manganese in Selected Organs of Adult C. gariepinus from Points A and C (mgkg^−1^)

	January	February	March	April	May	June	July	August	September

**Point A**									

G	0.45 ± 0.02a	0.50 ± 0.05a	0.47 ± 0.04a	0.43 ± 0.01a	0.38 ± 0.01a	0.38 ± 0.04a	0.30 ± 0.05a	0.21 ± 0.03a	0.19 ± 0.01a
H	0.28 ± 0.03b	0.33 ± 0.08bc	0.30 ± 0.02b	0.26 ± 0.02b	0.21 ± 0.05b	0.20 ± 0.02bc	0.10 ± 0.04b	0.08 ± 0.01b	0.08 ± 0.05b
B	0.33 ± 0.03b	0.38 ± 0.01b	0.36 ± 0.03b	0.31 ± 0.01b	0.31 ± 0.02b	0.32 ± 0.06b	0.14 ± 0.06b	0.16 ± 0.04b	0.10 ± 0.02b
M	0.18 ± 0.05c	0.26 ± 0.04c	0.21 ± 0.05c	0.18 ± 0.01c	0.13 ± 0.03c	0.10 ± 0.02c	0.06 ± 0.01c	0.02 ± 0.02c	0.02 ± 0.03c

**Point C**									

G	1.78± 0.26a	1.83 ± 0.24a	1.73 ± 0.44a	1.71 ± 0.25a	1.68 ± 0.32a	1.56 ± 0.42a	1.28 ± 0.24a	1.21 ± 0.60a	1.08 ± 0.42a
H	1.56 ± 0.18b	1.56 ± 0.12b	1.42 ± 0.10b	1.38 ± 0.10b	1.38 ± 0.24b	1.32 ± 0.22b	1.75 ± 0.46b	0.81 ± 0.15b	0.75 ± 0.23b
B	1.56 ± 0.11b	1.60 ± 0.44b	1.56 ± 0.26b	1.55 ± 0.24b	1.45 ± 0.21b	1.45 ± 0.16ab	1.46 ± 0.22b	0.90 ± 0.87b	0.83 ± 0.16b
M	1.42 ± 0.15c	1.44 ± 0.23c	1.32 ± 0.23c	1.26 ± 0.42c	1.23 ± 0.23c	1.19 ± 0.23c	1.81 ± 0.17bc	0.82 ± 0.22bc	0.71 ± 0.20c

Abbreviations: G, Gill; H, Head region; B, Bone; M, Muscle.

All data are mean ± standard deviation of triplicate determinations.

a, b, c indicate means from lowest to highest. Means in the same row with the same letter are not significantly different. Means within the same row with different letters are significantly different at p<0.05.

**Table 3:** Mean Monthly Value of Iron in Selected Organs of Adult C. gariepinus from Points A and C (mgkg^−1^)

	January	February	March	April	May	June	July	August	September

**Point A**									

G	1.31 ± 0.21a	1.31 ± 0.52a	1.20 ± 0.42a	1.18 ± 0.44a	1.15 ± 0.21a	1.08 ± 0.21a	1.00 ± 0.19a	0.96 ± 0.19a	0.83 ± 0.19a
H	1.20 ± 0.27b	1.24 ± 0.32b	1.10 ± 0.22b	1.06 ± 0.25b	0.96 ± 0.18b	0.83 ± 0.25b	0.72 ± 0.41b	0.65 ± 0.10b	0.58 ± 0.10b
B	1.25 ± 0.16a	1.26 ± 0.30ab	1.19 ± 0.15b	1.12 ± 0.12ab	0.10 ± 0.60b	0.10 ± 0.31ab	0.86 ± 0.10b	0.81 ± 0.15ab	0.58 ± 0.12b
M	1.16 ± 0.22b	1.19 ± 0.32c	0.88 ± 0.24c	0.88 ± 0.25c	0.69 ± 0.14c	0.51 ± 0.31c	0.46 ± 0.22c	0.41 ± 0.12c	0.32 ± 0.02c

**Point C**									

G	2.62 ± 0.05a	2.70 ± 1.13a	2.56 ± 1.13a	2.40 ± 1.21a	2.41 ± 1.31a	2.41 ± 1.02a	2.11 ± 0.98a	2.01 ± 1.08a	1.78 ± 1.13a
H	2.35 ± 0.08b	2.35 ± 1.12b	2.29 ± 1.15b	2.18 ± 1.03bc	2.18 ± 1.03c	2.11 ± 1.06c	1.81 ± 1.01b	1.62 ± 1.12b	1.53 ± 1.12b
B	2.38 ± 1.11b	2.42 ± 0.98b	2.38 ± 1.20b	2.33 ± 1.12b	2.29 ± 1.19b	2.31 ± 1.00b	1.92 ± 0.81b	1.77 ± 0.96b	1.60 ± 0.98b
M	2.26 ± 1.14c	2.30 ± 1.03bc	2.20 ± 0.96bc	1.96 ± l.00d	1.55 ± 1.12d	1.56 ± 0.91d	0.96 ± 0.99c	0.83 ± 1.03c	0.54 ± 1.03c

Abbreviations: G, Gill; H, Head region; B, Bone; M, Muscle.

All data are mean ± standard deviation of triplicate determinations.

a, b, c indicate means from lowest to highest. Means in the same row with the same letter are not significantly different. Means within the same row with different letters are significantly different at p<0.05.

**Table 4:** Mean Monthly Value of Lead in Selected Organs of Adult C. gariepinus from Points A and C (mgkg^−1^)

	January	February	March	April	May	June	July	August	September

**Point A**									

G	0.07 ± 0.02a	0.08 ± 0.01a	0.07 ± 0.03a	0.06 ± 0.02a	0.06 ± 0.01a	0.06 ± 0.01a	0.05 ± 0.01a	0.04 ± 0.00a	0.03 ± 0.01a
H	<0.01 ± 0.00b	<0.01 ± 0.00b	<0.01 ± 0.03b	<0.01 ± 0.00b	<0.01 ± 0.00b	<0.01 ± 0.00b	<0.01 ± 0.00b	<0.01 ± 0.00b	<0.01 ± 0.01b
B	<0.01 ± 0.00b	<0.01 ± 0.00b	<0.01 ± 0.03b	<0.01 ± 0.00b	<0.01 ± 0.00b	<0.01 ± 0.00b	<0.01 ± 0.00b	<0.01 ± 0.00b	<0.01 ± 0.01b
M	<0.01 ± 0.00b	<0.01 ± 0.00b	<0.01 ± 0.03b	<0.01 ± 0.00b	<0.01 ± 0.00b	<0.01 ± 0.00b	<0.01 ± 0.00b	<0.01 ± 0.00b	<0.01 ± 0.01b

**Point C**									

G	0.61 ± 0.01a	0.80 ± 0.03a	0.61 ± 0.01a	0.52 ± 0.05a	0.38 ± 0.01a	0.38 ± 0.04a	0.19 ± 0.05a	0.13 ± 0.01a	0.09 ± 0.02a
H	0.17 ± 0.00c	0.20 ± 0.01c	0.12 ± 0.02c	0.11 ± 0.02b	0.09 ± 0.05c	0.10 ± 0.02c	0.06 ± 0.01b	0.04 ± 0.00b	0.02 ± 0.01b
B	0.40 ± 0.01b	0.40 ± 0.01b	0.35 ± 0.01b	0.30 ± 0.03b	0.24 ± 0.02b	0.25 ± 0.06b	0.09 ± 0.02b	0.06 ± 0.00a	0.05 ± 0.01a
M	0.12 ± 0.01c	0.20 ± 0.00c	0.10 ± 0.01c	0.08 ± 0.01b	0.06 ± 0.03c	0.06 ± 0.02c	0.02 ± 0.01c	0.01 ± 0.00b	0.01 ± 0.01b

Abbreviations: G, Gill; H, Head region; B, Bone; M, Muscle.

All data are mean ± standard deviation of triplicate determinations.

a, b, c indicate means from lowest to highest. Means in the same row with the same letter are not significantly different. Means within the same row with different letters are significantly different at p<0.05.

**Table 5:** Mean Monthly Value of Copper in Selected Organs of Adult C. gariepinus from Points A and C (mgkg^−1^)

	January	February	March	April	May	June	July	August	September

**Point A**									

G	0.16 ± 0.03b	0.16 ± 0.01b	0.10 ± 0.01b	0.08 ± 0.03b	0.08 ± 0.01b	0.08 ± 0.01b	0.05 ± 0.01b	0.04 ± 0.02b	0.04 ± 0.01b
H	0.08 ± 0.01b	0.08 ± 0.02c	0.08 ± 0.01b	0.08 ± 0.01b	0.08 ± 0.02b	0.06 ± 0.00b	0.04 ± 0.01c	0.03 ± 0.01b	0.03 ± 0.02b
B	0.76 ± 0.01a	0.82 ± 0.01a	0.66 ± 0.02a	0.70 ± 0.06a	0.40 ± 0.01a	0.40 ± 0.00a	0.20 ± 0.00a	0.20 ± 0.05a	0.20 ± 0.08a
M	0.06 ± 0.00b	0.06 ± 0.01c	0.06 ± 0.03b	0.04 ± 0.01c	0.04 ± 0.01c	0.04 ± 0.01c	0.02 ± 0.01c	0.01 ± 0.01b	0.01 ± 0.01b

**Point C**									

G	1.08 ± 0.52b	1.11 ± 0.03a	0.80 ± 0.78a	0.60 ± 0.63b	0.40 ± 0.01b	0.40 ± 0.04b	0.30 ± 0.02b	0.26 ± 0.07b	0.12 ± 0.00b
H	0.12 ± 0.01c	0.13 ± 0.15b	0.14 ± 0.15b	0.12 ± 0.06c	0.01 ± 0.02c	1.10 ± 0.25c	0.08 ± 0.01c	0.07 ± 0.01c	0.04 ± 0.00c
B	1.23 ± 0.20a	1.16 ± 0.20a	1.20 ± 0.60a	1.20 ± 0.60a	1.00 ± 0.01a	1.00 ± 0.25a	0.95 ± 0.17a	0.90 ± 0.06a	0.60 ± 0.00a
M	0.08 ± 0.00c	0.09 ± 0.11b	0.10 ± 0.00b	0.10 ± 0.03c	0.12 ± 0.01c	0.09 ± 0.01c	0.07 ± 0.01c	0.06 ± 0.01c	0.04 ± 0.00c

Abbreviations: G, Gill; H, Head region; B, Bone; M, Muscle.

All data are mean ± standard deviation of triplicate determinations.

a, b, c indicate means from lowest to highest. Means in the same row with the same letter are not significantly different. Means within the same row with different letters are significantly different at p<0.05.

**Table 6:** Mean Monthly Value of Cadmium in Selected Organs of Adult C. gariepinus from Points A and C (mgkg^−1^)

	January	February	March	April	May	June	July	August	September

**Point A**									

G	<0.01 ± 0.00a	<0.01 ± 0.00a	<0.01 ± 0.00a	<0.01 ± 0.00a	<0.01 ± 0.00a	<0.01 ± 0.00a	<0.01 ± 0.00a	<0.01 ± 0.00a	<0.01 ± 0.00a
H	<0.01 ± 0.00a	<0.01 ± 0.00a	<0.01 ± 0.00a	<0.01 ± 0.00a	<0.01 ± 0.00a	<0.01 ± 0.00a	<0.01 ± 0.00a	<0.01 ± 0.00a	<0.01 ± 0.00a
B	<0.01 ± 0.00a	<0.01 ± 0.00a	<0.01 ± 0.00a	<0.01 ± 0.00a	<0.01 ± 0.00a	<0.01 ± 0.00a	<0.01 ± 0.00a	<0.01 ± 0.00a	<0.01 ± 0.00a
M	<0.01 ± 0.00a	<0.01 ± 0.00a	<0.01 ± 0.00a	<0.01 ± 0.00a	<0.01 ± 0.00a	<0.01 ± 0.00a	<0.01 ± 0.00a	<0.01 ± 0.00a	<0.01 ± 0.00a

**Point C**									

G	<0.01 ± 0.00a	<0.01 ± 0.00a	<0.01 ± 0.00a	<0.01 ± 0.00a	<0.01 ± 0.00a	<0.01 ± 0.00a	<0.01 ± 0.00a	<0.01 ± 0.00a	<0.01 ± 0.00a
H	<0.01 ± 0.00a	<0.01 ± 0.00a	<0.01 ± 0.00a	<0.01 ± 0.00a	<0.01 ± 0.00a	<0.01 ± 0.00a	<0.01 ± 0.00a	<0.01 ± 0.00a	<0.01 ± 0.00a
B	<0.01 ± 0.00a	<0.01 ± 0.00a	<0.01 ± 0.00a	<0.01 ± 0.00a	<0.01 ± 0.00a	<0.01 ± 0.00a	<0.01 ± 0.00a	<0.01 ± 0.00a	<0.01 ± 0.00a
M	<0.01± 0.00a	<0.01 ± 0.00a	<0.01 ± 0.00a	<0.01± 0.00a	<0.01 ± 0.00a	<0.01 ± 0.00a	<0.01 ± 0.00a	<0.01 ± 0.00a	<0.01 ± 0.00a

Abbreviations: G, Gill; H, Head region; B, Bone; M, Muscle.

All data are mean ± standard deviation of triplicate determinations.

a, b, c indicate means from lowest to highest. Means in the same row with the same letter are not significantly different. Means within the same row with different letters are significantly different at p<0.05.

**Table 7:** Mean Monthly Value of Chromium in Selected Organs of Adult C. gariepinus from Points A and C (mgkg^−1^)

	January	February	March	April	May	June	July	August	September

**Point A**									

G	<0.01 ± 0.00a	<0.01 ± 0.00a	<0.01 ± 0.00a	<0.01 ± 0.00a	<0.01 ± 0.00a	<0.01 ± 0.00a	<0.01 ± 0.00a	<0.01 ± 0.00a	<0.01 ± 0.00a
H	<0.01 ± 0.00a	<0.01 ± 0.00a	<0.01 ± 0.00a	<0.01 ± 0.00a	<0.01 ± 0.00a	<0.01 ± 0.00a	<0.01 ± 0.00a	<0.01 ± 0.00a	<0.01 ± 0.00a
B	<0.01 ± 0.00a	<0.01 ± 0.00a	<0.01 ± 0.00a	<0.01 ± 0.00a	<0.01 ± 0.00a	<0.01 ± 0.00a	<0.01 ± 0.00a	<0.01 ± 0.00a	<0.01 ± 0.00a
M	<0.01 ± 0.00a	<0.01 ± 0.00a	<0.01 ± 0.00a	<0.01 ± 0.00a	<0.01 ± 0.00a	<0.01 ± 0.00a	<0.01 ± 0.00a	<0.01 ± 0.00a	<0.01 ± 0.00a

**Point C**									

G	<0.04± 0.00a	<0.05± 0.00a	<0.02± 0.00a	<0.02± 0.00a	<0.02± 0.00a	<0.02± 0.00a	<0.01± 0.00a	<0.01± 0.00a	<0.01± 0.00a
H	<0.01± 0.00a	<0.01± 0.00a	<0.02± 0.00a	<0.02± 0.00a	<0.01± 0.00a	<0.01± 0.00a	<0.01± 0.00a	<0.01± 0.00a	<0.01± 0.00a
B	<0.04± 0.00a	<0.04± 0.00a	<0.04± 0.00a	<0.04± 0.00a	<0.04± 0.00a	<0.04± 0.00a	<0.01± 0.00a	<0.01± 0.00a	<0.01± 0.00a
M	<0.01± 0.00a	<0.02± 0.00a	<0.02± 0.00a	<0.02± 0.00a	<0.01± 0.00a	<0.01± 0.00a	<0.01± 0.00a	<0.01± 0.00a	<0.01± 0.00a

Abbreviations: G, Gill; H, Head region; B, Bone; M, Muscle.

All data are mean ± standard deviation of triplicate determinations.

a, b, c indicate means from lowest to highest. Means in the same row with the same letter are not significantly different. Means within the same row with different letters are significantly different at p<0.05.

The general level of copper concentrations in the tissues of the fish samples from Site A and C showed a different trend from other metals in the present study. The bone tissues accumulated high concentrations of metals followed by gill tissues > head regions > muscles. Site C had higher metal concentrations compared to Point A. The lowest metal concentrations were recorded in the month of September, while February had the highest concentrations. No fish were found at sample point B throughout the sampling period.

## Discussion

The Asa River supplies the bulk of water used by people in Ilorin and the surrounding area for a variety of activities, including laundry and recreation, cooling water for local industries and the river functions as a convenient point of waste discharge for the residential area and industry. However, this same water is dammed at some point, treated and distributed to serve the domestic needs of local residents.^1^ A previous study by Adekola and Eletta found that water and sediment in the Asa River are contaminated with metals.^1^ The range of concentration of these metals was Mn (179.9–469.4), Fe (1998.4–4420.4) Cr (3.0–11.3), Zn (26.6–147.6), and Cu (1.9–13.3) mg kg, above the World Health Organization (WHO) standards for water. The major sources of contamination can be traced to industrial discharge, domestic waste disposal and application of agrochemicals on farmland.

In fish, gills are considered to be the dominant site for contaminant uptake due to physiological properties that maximize absorption efficiency from water.^16^ The present study found that the gill was the site of maximum accumulation of heavy metal elements, while head and muscle showed the least metal accumulation. The higher levels of trace elements in gills relative to other tissues may be attributed to the affinity or strong coordination of metallothionein protein with these elements.^16^ However, gills and liver (although not inclusive in this study) are not commonly consumed, so consumption of fish collected from the river might not pose a serious health threat.

The significant differences in the bioaccumulation of metals in the tissues of African catfish from the two study sites along the Asa River and the seasonal variations found in the present study may be linked to observed behavioral differences and metabolic responses of fish to varying concentrations of effluent in the water, a factor that controls mortality as concentrations increase. Variations in the accumulation of metals in fish tissues could arise from factors which may be dependent on different ion accumulations in the species, season and fish habitat.^17^,^18^ A prominent trend in the bioaccumulation pattern of metals at points A and C was evident. African catfish collected from the two sampling sites accumulated high concentrations of zinc, manganese, iron, lead and chromium in the gills, followed by the bones, head regions and muscles, with the exception of copper, which showed high concentrations in bones, followed by gills, head regions and muscles. Clarias gariepinus was shown to bioaccumulate high concentrations of these metals at site C compared to site A. This trend further confirmed that point C may be an example of point pollution, while point A may demonstrate non-point pollution.

High concentrations of Zn, Mn, Fe, Pb, Cu and Cr were recorded in the tissues of C. gariepinus from the two study points in the month of February, a period that corresponded to the peak of the dry season, while low concentrations were recorded in the month of September, a period of flooding. These observations were in agreement with the findings of Coetzee et al., that C. gariepinus bioaccumulated high concentrations of metals in the months of February and May.^19^ Liver was shown to bioaccumulate high concentrations of metals. This observed trend could be linked to the absence of water dilution in the dry season, as the river experienced low flows during this period as a result of evaporation. The low values recorded in the month of September were probably due to dilution as a result of the large volume of water in the river during flooding.

Catfish gills, due to their proximity to the external environment and their ionic regulatory tendencies, could serve as a depot tissue, since metal uptake may exceed elimination. In freshwater fishes, the gills are the primary route for the uptake of waterborne pollutants, while the gut plays a secondary role. Zinc has two-fold influences on the gills, namely bioconcentration of metals and the structural cellular alterations as noted in the gills of Tilapia sparmanii. The high level of zinc in the gill tissues may be due to the fact that fish gills play a distinct role in metal uptake from the environment.

Concentrations of manganese and iron showed a similar sequence with that of zinc. Gills bioaccumulated high concentrations of manganese and iron, followed by bone, head region and muscles (G > B >H > M). This is similar to the assertion of Coetzee et al., that the high manganese concentration in the gills could be attributed to the role of the gills as the main route of uptake of manganese as little absorption of this metal occurs through the gut from food.^19^ High iron concentrations in the gill tissues may be due to iron-containing enzymes and the extensive vascular system of the gill, as hemoglobin in the blood binds approximately three quarters of the iron in the body.

Muscle accumulated only small amounts and similar concentrations of lead. Fishes are said to accumulate very little lead in edible tissues. Low concentrations of lead in the head region and bone could be linked to the low binding rate of lead to sulphydryl groups.^20^ Lead could possibly interfere with the action of gonadotrophins by binding to the sulphydryl groups on the cell membrane receptor sites for these molecules.

The levels of copper in the bone in the present study were usually slightly higher than those found in the gills, tissues, head region and bone. This is in contrast with the trend and observation of Seymore et al. and Allen et al. that gills are target organs for copper toxicity in fish and there is usually a positive correlation between copper concentrations in gill tissues and the environment.^21^,^22^ The trend (bone > gills > head region and muscle) could be due to the absorption properties of copper into suspended particles and skeletal parts.

All of the examined tissues in the present study showed insignificant concentrations of Cd and Cr. This is similar to the observations of Allen et al.^22^ and could be ascribed to variations in the chemical form of Cd and Cr, differences in exposure period, and varied tissue composition. The observation could also be a result of alteration in the morphology of the gills and other tissues, leading to a reduction in Cd and Cr binding capacity, leading to low accumulations of Cd and Cr. Due to fluctuating Cd and Cr levels in the water, fish do not experience mass cumulative Cd and Cr uptake, as these metals are rapidly eliminated by fish in water with low concentrations or in non-contaminated water.^23–27^

## Conclusions

The present study demonstrated that the prevailing practice of unregulated and uncontrolled discharge of detergent effluent into water bodies poses a serious threat to aquatic organisms. The bioaccumulation level of heavy metals in selected organs and tissues of African catfish in Asa River, Ilorin, Kwara State, Nigeria was very high and the level of accumulation of heavy metals increased downstream. Accumulations were much higher during the dry season (especially in the month of February) and relatively low during the peak of the rainy season. Heavy metals at elevated levels in the aquatic environment can bioaccumulate in fish tissues, and therefore fish from the Asa River are considered unfit for human consumption. It is therefore recommended that the Asa River should be put under surveillance since it is the only source of fresh water in this area.
